# Gene rearrangement and Chernobyl related thyroid cancers

**DOI:** 10.1054/bjoc.1999.0921

**Published:** 2000-01-18

**Authors:** M Santoro, G A Thomas, G Vecchio, G H Williams, A Fusco, G Chiappetta, V Pozcharskaya, T I Bogdanova, E P Demidchik, E D Cherstvoy, L Voscoboinik, N D Tronko, A Carss, H Bunnell, M Tonnachera, J Parma, J E Dumont, G Keller, H Höfler, E D Williams

**Affiliations:** Centro di Endocrinologia ed Oncologia Sperimentale del CNR c/o Dipartimento di Biologia e Patologia Cellulare, e Moleculare, Universita di Napoli, Italy; Thyroid Carcinogenesis Group, University of Cambridge, Strangeways Research Laboratory, Wort's Causeway, Cambridge, CB1 4RN, UK; Minsk State Medical Institute, Minsk, Belarus; Institute of Endocrinology and Metabolism, Kiev, Ukraine; IRIHBN, Free University of Brussels, Belgium; Institute for Pathology, Technical University of Munich, Germany

**Keywords:** thyroid cancer, Chernobyl, gene rearrangement, ref, oncogenes

## Abstract

The increase in thyroid carcinoma post-Chernobyl has been largely confined to a specific subtype of papillary carcinoma (solid/follicular). This subtype is observed predominantly in children under 10 in unirradiated populations, but maintains a high frequency in those aged 10–15 from those areas exposed to fallout from the Chernobyl accident. The aim of this study was to link morphology with molecular biology. We examined 106 papillary carcinomas from children under the age of 15 at operation. All were examined for rearrangements of the *RET* oncogene by reverse transcription polymerase chain reaction (RT-PCR); a subset of these cases were also examined for mutations of the three ras oncogenes, exon 10 of the thyroid stimulating hormone receptor, associated more usually with a follicular rather than papillary morphology, and exons 5, 6, 7 and 8 of the p53 gene, commonly involved in undifferentiated thyroid carcinoma. Rearrangements of the *RET* oncogene were found in 44% of papillary carcinomas in which we studied fresh material; none of the tumours examined showed mutation in any of the other genes. The two rearrangements resulting from inversion of part of chromosome 10 (PTC1 and PTC3) accounted for the majority of *RET* rearrangements identified, with PTC1 being associated with papillary carcinomas of the classic and diffuse sclerosing variants and PTC3 with the solid/follicular variant. © 2000 Cancer Research Campaign


*British Journal of Cancer* (2000) **82**(2), 315–322
© 2000 Cancer Research Campaign
Article no. bjoc.1999.0921

### Gene rearrangement and Chernobyl related thyroid cancers

**M Santoro[1], GA Thomas[2], G Vecchio[1], GH Williams[2], A Fusco[1], G Chiappetta[1], V Pozcharskaya[3], TI Bogdanova[4], EP Demidchik[3], ED Cherstvoy[3], L Voscoboinik[4], ND Tronko[4], A Carss[2], H Bunnell[2], M Tonnachera[5], J Parma[5], JE Dumont[5], G Keller[6], H Höfler[6] and ED Williams[2]**

[1]Centro di Endocrinologia ed Oncologia Sperimentale del CNR c/o Dipartimento di Biologia e Patologia Cellulare e Moleculare, Universita di Napoli, Italy; [2]Thyroid Carcinogenesis Group, University of Cambridge, Strangeways Research Laboratory, Wort's Causeway, Cambridge CB1 4RN, UK; [3]Minsk State Medical Institute, Minsk, Belarus; [4]Institute of Endocrinology and Metabolism, Kiev, Ukraine; [5]IRIHBN, Free University of Brussels, Belgium, [6]Institute for Pathology, Technical University of Munich, Germany

**Summary** The increase in thyroid carcinoma post-Chernobyl has been largely confined to a specific subtype of papillary carcinoma (solid/follicular). This subtype is observed predominantly in children under 10 in unirradiated populations, but maintains a high frequency in those aged 10–15 from those areas exposed to fallout from the Chernobyl accident. The aim of this study was to link morphology with molecular biology. We examined 106 papillary carcinomas from children under the age of 15 at operation. All were examined for rearrangements of the *RET* oncogene by reverse transcription polymerase chain reaction (RT-PCR); a subset of these cases were also examined for mutations of the three ras oncogenes, exon 10 of the thyroid stimulating hormone receptor, associated more usually with a follicular rather than papillary morphology, and exons 5, 6, 7 and 8 of the p53 gene, commonly involved in undifferentiated thyroid carcinoma. Rearrangements of the *RET* oncogene were found in 44% of papillary carcinomas in which we studied fresh material; none of the tumours examined showed mutation in any of the other genes. The two rearrangements resulting from inversion of part of chromosome 10 (PTC1 and PTC3) accounted for the majority of *RET* rearrangements identified, with PTC1 being associated with papillary carcinomas of the classic and diffuse sclerosing variants and PTC3 with the solid/follicular variant. © 2000 Cancer Research Campaign

**Keywords**: thyroid cancer; Chernobyl; gene rearrangement; ref; oncogenes

The accident at the Chernobyl nuclear power plant on 26 April 1986 resulted in the population of Southern Belarus and Northern Ukraine being exposed to radioactive fallout which contained substantial amounts of radioactive iodine. It became apparent in 1992 that there was an increase in childhood thyroid carcinoma (Baverstock et al, 1992; Kazakov et al, 1992). Thyroid carcinoma in children is usually rare (of the order of 0.3–1.3 million$^{-1}$ year$^{-1}$; Parkin et al, 1988). The first reaction to the increase in thyroid carcinoma was one of surprise, due largely to the reported size of the increase and the short latent period. However, a number of published studies support the view that the tumours are causally related to exposure from Chernobyl (Likhtarev et al, 1995; Williams, 1996; Jacob et al, 1998).

Two morphologically distinct types of differentiated carcinoma derive from the thyroid follicular cell, papillary carcinoma and follicular carcinoma. The two different types exhibit not only different morphology but also different biological behaviour and are associated with mutations in different oncogenes; papillary carcinoma with rearrangement of either the *RET* or trk oncogenes (Greco et al, 1992; Santoro et al, 1992) and follicular carcinoma with mutation of the ras oncogene (Lemoine et al, 1989). Mutations of exon 10 of the thyroid stimulating hormone (TSH) receptor have been particularly associated with a specific type of benign thyroid tumour, the hot nodule (Parma et al, 1995), but there have been reports of TSH receptor mutation in follicular carcinoma (Russo et al, 1995). The p53 oncogene is commonly mutated in many different types of carcinoma; in thyroid carcinoma it is associated with anaplastic carcinoma, rather than the differentiated types of thyroid carcinoma (Ito et al, 1992).

The majority of the rise in post-Chernobyl thyroid carcinoma incidence has been due to an increase in papillary carcinoma. However, papillary carcinoma in children can be subdivided into three main subtypes, solid/follicular, classical and diffuse sclerosing variant (Harach and Williams, 1995). The morphological characteristics of any tumour result from the integration of the molecular biological changes involved in the genesis of that tumour. In this study we have therefore correlated the morphological findings with molecular biological analysis of mutation in four oncogenes associated with thyroid carcinogenesis (*RET*, ras, p53 and exon 10 of the TSH receptor).

*Received 24 March 1999*
*Revised 22 July 1999*
*Accepted 25 August 1999*

*Correspondence to:* GA Thomas

### METHODS

### Verification of pathology

All material was provided by the Institute of Pathology in Minsk and the Institute of Endocrinology and Metabolism in Kiev. Sections from representative paraffin blocks from each of the cases examined were stained with haematoxylin and eosin (H&E), and with a standard immunocytochemical techique for calcitonin and thyroglobulin. Papillary carcinomas were classified as either solid/follicular, classical or diffuse sclerosing variant based on their dominant morphological component, using definitions given in our previous study of the morphology of childhood thyroid cancer in England and Wales (Harach and Williams, 1995). Where possible, frozen sections of unfixed material for DNA and RNA extraction were also examined to determine the presence and amount of tumour epithelium in the frozen material.

A total of 132 cases were used for this molecular biological study. Fresh material was available in 50 cases; analyses were performed on paraffin sections for the remaining 82 cases. All cases were operated in Minsk or Kiev between 1990 and 1996. Serial paraffin sections were cut at one centre and circulated to the other centres for extraction and analysis of the various oncogenes. Paraffin material was only available from those cases operated in 1990–1994, fresh material was available from cases operated in 1995 and 1996. DNA and RNA from fresh material was extracted at one centre using a standard Trizol™ protocol. Aliquots were distributed to other centres for analysis of *RET* rearrangement, mutation of the TSH receptor, Ki, Ha, N ras and p53 oncogenes.

### RT-PCR for RET/PTC expression

Reverse transcription and subsequent PCR amplification was performed on both paraffin and fresh material as previously reported. The sequence of the forward primers used were: for *RET*/PTC1: 5′-ATTGTCATCTCGCCGTTC-3′, specific for the H4 gene, and for *RET*/PTC3: 5′-AAGCAAACCTGCCAGTGG-3′, specific for the *ele1* gene. The sequence of the reverse primer was: 5′-TGCTTCAGGACGTTGAAC-3′, specific for *RET* tyrosine kinase. One microgram of RNA was reverse transcribed using the reverse primer and, following the addition of the forward primer, subjected to 40 cycles of PCR (94°C for 30 min, 55°C for 2 min and 72°C for 2 min). The product of the reaction was analysed on a 2% agarose gel and hybridized with a probe for the *RET* tyrosine-kinase domain. The *HPRT* specific primers were: 5′-CCTGCTG-GATTACATCAAAGCACTG-3′ (forward), corresponding to the nucleotides 316–340 of the third exon of the human gene; 5′-CCTGAAGTATTCATTATAGTCTCAAGG-3′ (reverse) corresponding to the nucleotides 685–661 of the eighth exon of the human gene.

### RT-PCR method for detecting expression of the *RET* tyrosine kinase domain (TK) and extracellular domain (EC)

The reverse transcription reaction was performed on 1 µg of RNA. The RT reaction was performed in a final volume of 25 µl containing 0.1 optical density units of random hexamers (Pharmacia P(N)6), 2.5 µl of 10× RT buffer (1×RT 50 mM Tris–HCl (pH 8.3), 50 mM potassium chloride (KCl), 4 mM dithiothreitol (DTT), 10 mM magnesium chloride ($MgCl_2$)), 2.5 µl of 10 mM dNTPs, 1 unit µl$^{-1}$ RNasin and 200 units ml$^{-1}$ of super RT and incubated at 41°C for 60 min. The reaction was terminated at 95°C for 5 min. Fifteen microlitres of the reaction mixture were used for PCR amplification with primers 5′-CACCGGATGGA-GAGGCCAGACAACTGCAGC-3′ and 5′-ACCGGCCTTTTGT-CCGGCTC-3′ (TK), and5′-GTGCAGTTCTTGTGCCCCAAC-ATCAG-3′ and 5′-CCCAGCGCGTGCTCACCT-3′ (extracellular) in a final volume of 100 µl. Primers for the 3′ end of the *RET* oncogene (TK) corresponded to exon 16, 5′ upstream and exon 17, 3′ downstream spanning a 1150 bp intron, primers for the extracellular domain were designed to span intron 4, giving a product from RNA of 106 bp. PCR reactions consisted of 35 cycles of amplification at 95°C for 30 s, 50°C for 30 s and 72°C for 1 min. Precautions were taken to prevent PCR contamination. Parallel reagent negative controls were run with all samples to exclude generalized PCR contamination. 15 µl of each PCR reaction was run on a 2% agarose gel together with ØX markers. The specificity of the PCR product was confirmed by Southern blotting using a probe specific for 3′ region of the *RET* gene.

S6 ribosomal mRNA was used as a reporter target for amplification to monitor the presence of RNA; a negative amplification result indicated RNA degradation. The primers 5′-AATCCG-CAAACGTTTCAATCTCT-3′ and 5′-TGAATCTTGGGTGCTT-TGGTCCTA-3′ were chosen in different exons to generate a 106 bp PCR product from mRNA, readily distinguished from S6 genomic amplification which would result in a PCR product of 1826 bp. Amplification was performed for 35 cycles using the same conditions as for *RET* TK and extracellular PCRs. RNA extracted from the SHSY neuroblastoma cell line, which expresses c-*RET*, was used as a positive control. RNA extracted from the 293 kidney cell line, which does not express c-*RET* was used as a negative control, together with conventional no template controls. Both cell lines were kindly provided by Professor Bruce Ponder (Human Cancer Genetics Group, University of Cambridge).

### TSH receptor mutation

Paraffin sections were washed in xylene and ethanol. After vacuum drying of the pellet, proteinase K (1 mg ml$^{-1}$) in 50 mM Tris buffer was added at 55°C for 2 h and then at 37°C overnight. After heating the sample for 10 min at 100°C to inactivate the proteinase K, the quality of the DNA was checked on a 0.8% agarose gel, which showed the presence of high molecular weight DNA after ethidium bromide staining.

Two fragments (bases 2078–2664 and bases 1273–2340) of the TSH receptor gene were amplified by PCR and subjected to double-strand direct sequencing. The antisense primer was biotinylated in each case. The PCR reaction was performed in a final volume of 100 ml, containing 2 ml of DNA, 50 mM KCl, 10 mM Tris–HCl pH 8.3, 1.5 mM $MgCl_2$, 0.01% gelatin, 0.2 mM dNTP, 5 U *Taq* polymerase (Boehringer Mannheim, Germany) and 200 nM of each primer. Samples were denatured at 93°C for 2 min for the first pair of primers followed by 50 cycles of 93°C for 1 min, 54°C for 1 min and 72°C for 2 min 30 s. A final extension of 6 min at 72°C was performed. The protocol for the second pair of primers differed by using an annealing temperature of 50°C rather than 53°C. PCR products were visualized by ethidium bromide staining on agarose gels; a strong band of the expected size (600 bp) was observed by using the first pair of primers, while a faint band of the expected size (1000 bp) was observed with the second pair of primers. PCR negative control (water instead of DNA template) and the material obtained by using a paraffin block devoid of tissue did not show any amplification. Mutations in the TSH receptor amplimers were identified by single-strand conformation polymorphism (SSCP), and confirmed by sequencing.

For DNA from frozen specimens, four fragments of the TSH receptor gene were amplified by PCR and subjected to double-strand direct sequencing. The fragments and the primers used

*© 2000 Cancer Research Campaign*

were: A; residues 235–294, forward primer 5′-M13D TCA TCT CCC AAT TAA CCT CAG G-3′; reverse primer 5′-T7 GCT TCC AAT TTC CTC TCC AC-3′; fragment B, residues 430–537 forward primer 5′-T7 TTC GTT AGT CTG CTG GCT C-3′, reverse primer 5′-M13D CAA CCA TGA TGG CAC ATG-3′; fragment C: residues 526–646, forward primer 5′-M13D CTG GTA TGC CAT CAC CTT-3′, reverse primer 5′-M13R TGA GAG GCT TGT TCA GAA TT-3′; fragment D: residues 635–stop forward primer 5′-M13D TGT TGA TCT TCA CCG ACT TC-3′, reverse primer 5′-M13R TAA GTT CCC CTA CCA TTG TG-3′ {M13D, M13R and T7 correspond to universal sequencing primers (ABI)}. Standard PCR was carried out in a 20 µl volume containing 200 ng DNA, 50 mM KCl, 10 mM Tris–HCl, pH 8.3, 2 mM for fragment A, or 1 mM for fragment B, C and D of $MgCl_2$, 0.01% gelatine, 0.2 mM dNTP, 5 U *Taq* polymerase (BRL) and 300 nM for fragment A, 150 nM for fragment B and D or 100 nM for fragment C of each primer. The annealing temperatures were 61°C for fragment A, 52°C for fragments B, 50°C for C and 48°C for D respectively. Direct genomic DNA sequencing of both strands was realized with the dye primer cycle sequencing core kit (ABI: PN 402070) and mutations searched for by Factura and Sequence Navigator Software running on an ABI 373 sequencer.

### Ras gene mutation

Tumours were microdissected from paraffin-embedded sections. Following digestion with proteinase K (200 mg $ml^{-1}$) at 55°C overnight, PCR was performed in a final volume of 50 µl containing 50 mM KCl, 10 mM Tris–HCl (pH 8.4), 100 g $ml^{-1}$ gelatin, 0.2 µM of each primer, 200 µmol of each of dATP, dCTP, dGTP and dTTP and 2.5 units of *Taq* polymerase. Exons 1 and 2 of each of the 3 *ras* genes (Ha, Ki and N) were amplified. The primer pairs used and PCR conditions for each gene are given in Table 1. Following a hot start of 95°C for 3 min, 35 cycles of PCR were performed consisting of 95°C for 30 s, 48–50°C for 30 s and 72°C for 1 min followed by a final extension step of 72°C for 6 min. One of each of the primer pairs was biotinylated; direct sequencing of the product was carried out using the Dynal and Sequenase 2.0 kits.

### p53 mutation analysis

Tissue was microdissected from tumour areas with at least 50% tumour. Paraffin-embedded tissues were dewaxed with xylene, washed with ethanol, vacuum-desiccated, resuspended in 200 µl 50 mmol $l^{-1}$ Tris–HCl pH 8.5, 1 mmol $l^{-1}$ EDTA, 0.5% Tween-20, 0.2 mg $ml^{-1}$ proteinase K and incubated at 55°C for at least 3 h. Proteinase K was inactivated by boiling for 10 min. Two microlitres of 1:10 dilution were directly used for PCR.

The most conserved regions of the p53 gene comprising exons 5–8 were amplified by PCR as described (Lohmann et al, 1993) with the exception that exons 6 and 8 were amplified without nested PCR using only the external primers for the corresponding exons.

PCR products were diluted 1:2 with 95% formamide, 10 mM sodium hydroxide (NaOH) 0.05% xylene cyanol, 0.05% bromophenol blue, denatured at 95°C for 5 min and chilled on ice. Electrophoresis was performed for each exon under four different running conditions including those previously found to give optimal resolution determined from known p53 mutations which had been identified by direct sequencing (Lohmann et al, 1993). In brief, 5 µl of denatured PCR product were loaded on a horizontal 8% polyacrylamide gel (30:05) containing 2% glycerine in a 20 mM MOPS (pH 8.0), 1 mM EDTA running buffer (TGGE system, Diagen, Düsseldorf, Germany). In addition, electrophoresis was performed in a discontinuous borate-phosphate-buffer system using 90 mM Tris–borate as running buffer and 8% polyacrylamide gels containing 2% glycerol prepared in a 90 mM Tris–phosphate buffer (pH 8.0). Electrophoresis was performed at 15°C and 25°C for exons 5–8 and at 5°C and 15°C for exon 6. Visualization of DNA was performed by silver staining. Gels were washed and fixed in 10% ethanol, 0.5% acetic acid for 10 min, stained with 0.1% $AgNO_3$ for 10 min, washed in distilled water and developed in 1.5% NaOH, 0.15% formaldehyde and 0.01% $NaBH_4$.

Analysis of p53 mutation and mutation in the ras oncogenes were performed on paraffin material only; analysis of *RET* expression was performed on frozen material only.

### RESULTS

The majority (128/132) of the cases studied were papillary carcinomas, three were follicular carcinomas and one was a medullary carcinoma. The majority (74%) of the papillary carcinomas were of the solid/follicular subtype, which is the commonest subtype of papillary carcinoma post-Chernobyl (Bogdanova et al, 1996; Cherstvoy et al, 1996; Williams, 1996) (Table 2). Of the 106 papillary carcinomas which produced amplifiable RNA and were analysed for *RET* rearrangement using rearrangement specific RT-PCR, 20 were identified as positive for PTC1 and 15 for PTC3; one other tumour was positive for both PTC1 and PTC3. Examples of these results are given in Figures 1 and 2. Analysis of the expression of the extracellular and tyrosine kinase domains was also performed independently in a different institute in 45 of the 106 cases. Twenty-two of these 45 tumours were positive for expression of *RET* tyrosine kinase. No tumour sample showed positivity for the expression of the *RET* extracellular domain, suggesting that the

**Table 1** Primer pairs and PCR conditions for amplification of ras oncogenes

| Region | Sense 5′–3′ | Antisense 5′–3′ | Annealing temperature | $MgCl_2$ concn. |
|---|---|---|---|---|
| K ras exon 1 | GACTGAATATAAACTTGTGG | CTATTGTTGGATCATATTCG | 52ºC | 1.5 mM |
| K ras exon 2 | AAGTAGTAATTGATGGAGAA | CATGTACTGGTCCCTCATT | 52ºC | 2.5 mM |
| H ras exon 1 | GACGGAATATAAGCTGGTGGTG | CTATAGTGGGGTCTGATTCG | 48ºC | 1.5 mM |
| H ras exon 2 | AGGTGGTCATTGATGGGGAG | CATGTACTGGTCCCGCATG | 48ºC | 1.5 mM |
| N ras exon 1 | ATGACTGAGTACAAACTGGT | CTATGGTGGGATCATATTCA | 50ºC | 1.5 mM |
| N ras exon 2 | AGGTGGTTATAGATGGTGAA | CATGTATTGGTCTCTCATG | 48ºC | 1.5 mM |

**Table 2** Histology of post-Chernobyl thyroid carcinomas examined for mutation of various oncogenes

| Type of material | Fixed material only | Frozen material |
|---|---|---|
| Total number of tumours | 82 | 50 |
| No. papillary carcinomas | 80 | 48 |
| No. not analysable[a] | 19 | 3 |
| No. analysed | 61 | 45 |
| *Subtype of papillary carcinoma in the tumours analysed* | | |
| Solid/follicular | 46 (75%) | 32 (71%) |
| Classic or diffuse sclerosing variant | 15 (25%) | 11 (25%) |
| Insufficient to subclassify | 0 | 2 (4%) |

[a] Nucleic acid of inadequate quality.

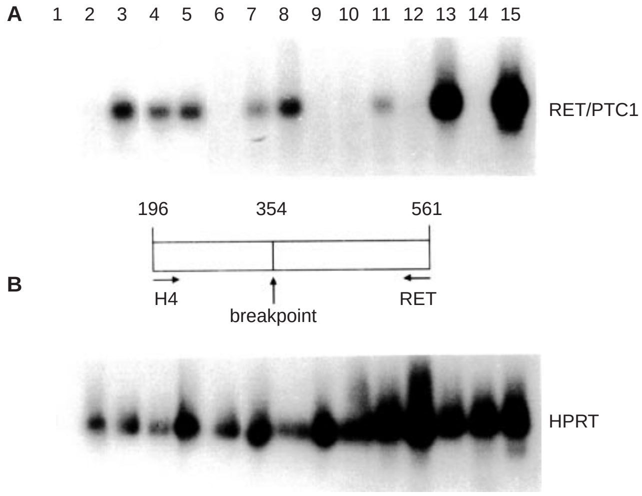

**Figure 1** RET/PTC1 detection in Chernobyl papillary thyroid carcinomas. (**A**) RNAs were amplified with *RET*/PTC1-specific primers, as indicated in the schematic diagram, subjected to electrophoretic separation on a 2% agarose gel, and hybridized with a *RET* probe covering its TK domain. Lane 1: a negative control represented by RNA from the TPC cell line (a human papillary carcinoma cell line spontaneously expressing a RET/PTC1 oncogene) subjected to PCR without previous reverse-transcription. Lane 2: another negative control represented by normal thyroid RNA; lanes 3–14: representative examples of RNAs from papillary thyroid carcinomas from the Chernobyl area showing seven positive samples. Amplification of the RNAs without previous reverse transcription gave negative results (data not shown). Lane 15: a positive control represented by RNA extracted from the TPC cell line. (**B**) The same RNAs were subjected to RT-PCR amplification using HPRT (human phosphoribosyl transferase)-specific primers. The products of the amplification were run on a 2% agarose gel and hybridized to an HPRT-specific probe. This confirmed that the negative results were not due to absence of amplifiable RNA

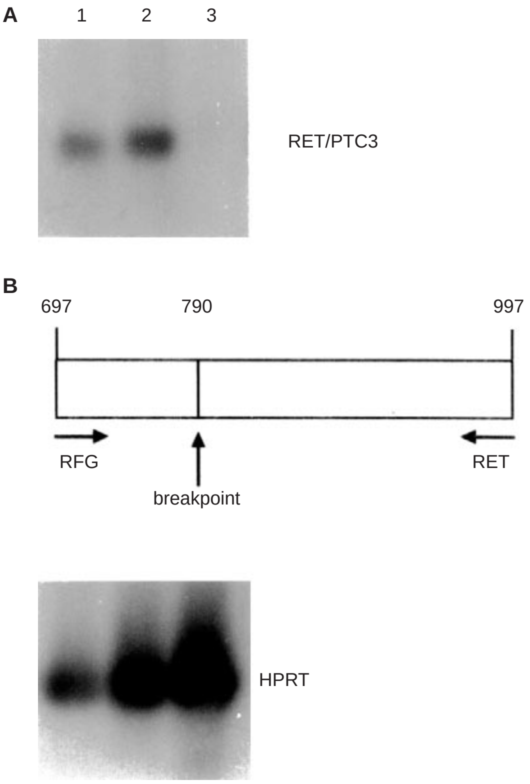

**Figure 2** RET/PTC3 detection in Chernobyl papillary thyroid carcinomas. (**A**) RNAs were amplified with *RET*/PTC3 specific primers, as indicated in the schematic diagram, subjected to electrophoretic separation on a 2% agarose gel, and hybridized with a *RET* probe covering its TK domain. Lanes 1–2: two representative cases scored positive for RET/PTC3 expression. Lane 3: a negative control represented by normal thyroid RNA. (**B**) The same RNAs were subjected to RT-PCR amplification using HPRT (human phosphoribosyl transferase)-specific primers. The products of the amplification were run on a 2% agarose gel and hybridized to an HPRT-specific probe

*RET* tyrosine kinase expression was due to the presence in these tumours of a *RET* rearrangement fusing the *RET* tyrosine kinase domain of the c-*RET* gene with an active promoter. Among these 45 cases investigated for *RET* tyrosine kinase expression, there were 20 with an identified PTC1 or -3 rearrangement, including one tumour which showed both PTC1 and -3; 18 showed *RET* tyrosine kinase expression. The unexpected negative *RET* tyrosine kinase in two cases may be the result of a lack of sensitivity. Twenty-five cases lacked either a PTC1 or -3 rearrangement, 21 were negative for *RET* tyrosine kinase expression, while four were positive. The positive *RET* tyrosine kinase in these four cases could result from the presence of a *RET* rearrangement other than PTC1 or -3, for example those described by Fugazzola et al (1995) and Klugbauer et al (1995, 1998). Overall agreement between the two techniques was therefore present in 39 out of 45 cases (87%).

We have compared the frequency of detection of *RET* rearrangement in formalin-fixed paraffin-embedded material with that from fresh tissue, but due to availability of material were only able to do that by comparing the frequency in different groups of tumours, not in identical tumours (Table 3). Adequate RNA was available

 © 2000 Cancer Research Campaign

**Table 3** Identification of PTC1 or -3 rearrangement of the ret oncogene from formalin-fixed, paraffin-embedded and frozen material

| Type of material | Fixed material (cases operated 1990–1994) | Frozen material (cases operated 1995–1996) |
|---|---|---|
| No. papillary carcinomas | 80 | 48 |
| No. not analysable[a] | 19 | 3 |
| No. analysed | 61 | 45 |
| PTC1 | 13 | 7 |
| PTC3 | 3 | 12 |
| PTC1 and PTC3 | 0 | 1 |
| Negative for PTC1 or -3 | 45 | 25 |

[a] RNA of inadequate quality.

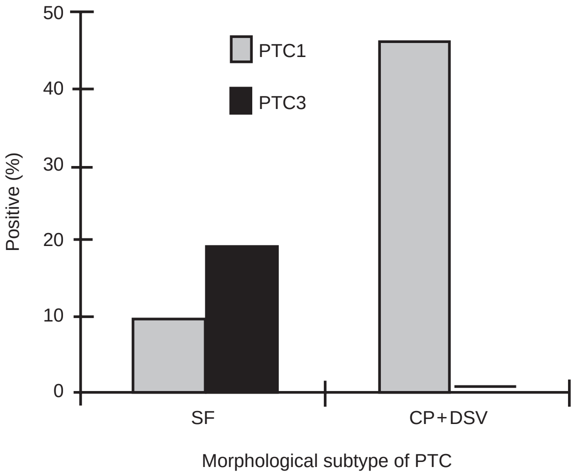

**Figure 3** Association of different ret rearrangements with morphological subtypes of papillary carcinoma

**Table 4** Oncogene involvement in post- Chernobyl papillary carcinomas

| | No. studied | No. positive | No. negative | % positive |
|---|---|---|---|---|
| ret rearrangement | 106 | 36 | 70 | 34 |
| ret TK expression | 46 | 23 | 23 | 50 |
| TSHR | 57 | 0 | 57 | 0 |
| p53 | 35 | 0 | 35 | 0 |
| Ki | 31 | 0 | 31 | 0 |
| N | 23 | 0 | 23 | 0 |
| Ha | 23 | 0 | 23 | 0 |

Rearrangement of the ret oncogene was assessed in two ways; by identification of the PTC1 and PTC3 rearrangement and also by expression of the 3′ end of the c-ret gene in the absence of expression of the extracellular domain. Exon 10 of the TSH receptor, exons 5, 6, 7 and 8 of the p53 gene, together with codons 12, 13 and 61 of the ras oncogenes were examined for mutation. Ret rearrangement was analysed on both frozen (cases operated in 1995–96) and paraffin material (cases operated in 1990–4). Ret TK expression was carried out on frozen material only. TSHR mutation was carried out on a subset of both paraffin and frozen material. The remaining oncogenes were examined in paraffin specimens only.

from paraffin sections of 61 tumours resected from 1990 to 1994. Sixteen were positive for *RET* rearrangement (13 PTC1 and three PTC3). Adequate RNA was available from fresh tissue from 45 cases resected in 1995–1996; these are the same group in which *RET* TK expression was compared with *RET* rearrangement. Of these 45 cases, 20 showed *RET* rearrangement, eight PTC1 and 13 PTC3 (one tumour had both rearrangements).

The type of *RET* rearrangement correlated with the morphological type of papillary carcinomas, with PTC3 being found more commonly in those of solid/follicular morphology, and PTC1 found in the less common classic papillary and diffuse sclerosing variant of papillary carcinoma (see Figure 3).

No mutations in any of the *ras* genes, exon 10 of the TSH receptor or in exons 6, 7 or 8 of the p53 gene were identified in any tumour (Table 4).

### DISCUSSION

We present the results of a comprehensive study of a large series of post-Chernobyl papillary carcinomas studying a range of different types of oncogenes in a large series of tumours, and comparing these findings to detailed morphology.

*RET* gene rearrangements have been reported in about one-third of adult papillary carcinomas not known to be radiation related, but were not found in any other type of thyroid carcinoma (Santoro et al, 1992). One study of thyroid tumours in adult patients with a history of external radiation found that ret rearrangements occurred in follicular adenomas as well as papillary carcinomas (Bounacer et al, 1997). A more recent study, which reported findings on children exposed to fallout from the Chernobyl accident found that ret rearrangement was restricted to papillary carcinomas; no rearrangement of this gene was observed in follicular tumours (Thomas et al, 1999). In the majority of studies of tumours not known to be related to radiation exposure, ret rearrangement has been found only in papillary carcinomas. In a study of 21 cases of papillary carcinoma from children under 15 at operation in England and Wales 47% showed expression of the *RET* oncogene suggesting the presence of a rearrangement (Williams et al, 1996). The relative frequency of the two major subtypes of ret rearrangement (PTC1 and -3) varies in studies which report their incidence post-Chernobyl. In two small studies of Chernobyl-related thyroid cancer, one with four cases with rearrangement and one with nine, PTC3 was the commonest type of *RET* rearrangement found (Fuggazola et al, 1995; Klugbauer et al, 1995). In a larger study, 33 of 38 selected cancers from patients under the age of 18 from Belarus showed a *RET* rearrangement, 22 of these were PTC3 (Nikiforov et al, 1997). In the most recent reported study of 51 cases under 16 at operation, 25 papillary carcinomas showed a ret rearrangement, with an almost equal frequency of the two types of rearrangement (Smida et al, 1999). We have found both PTC1 and -3 in these 106 cases, all under 15 at operation, but have also shown that the two commonest *RET*

*© 2000 Cancer Research Campaign* 

rearrangements, PTC1 and -3, show an association with different morphologies of papillary carcinoma, PTC1 being more common in tumours with a classical or diffuse sclerosing phenotype and PTC3 more common in papillary carcinomas with a solid/-follicular phenotype ($P < 0.001$ $\chi^2$ test), in agreement with and extending the earlier conclusions of Nikiforov et al (1997) who showed that papillary tumours with a solid architecture from exposed children had a high prevalence of PTC3 rearrangement. This correlation might be related to the level of *RET* TK expression in the two rearrangements, or to an influence of the gene to which the *RET* TK is fused, or to an effect related to the reciprocal breakpoint. However, transgenic mice which have a thyroid targetted expression of the PTC1 or -3 rearrangement also show the same association between morphology and expression of the different *RET* rearrangements, with PTC1 transgenic animals showing tumours of classical papillary morphology (Jhiang et al, 1996; Santoro et al, 1996) and PTC3 transgenic mice showing tumours of predominantly solid morphology (Powell et al, 1998). The finding that the inappropriate expression of the same tyrosine kinase in these animals results in an alteration of the morphology of the tumours produced suggests a morphogenetic role for the gene to which the *RET* tyrosine kinase is fused.

The differing frequencies of *RET* rearrangement found in the series of cases which we analysed on paraffin and the series analysed from frozen cannot be explained by differing frequencies of papillary carcinomas with solid/follicular morphology within the two series; 75% of the papillary carcinomas analysed were of this morphology in the paraffin series and 71% in the frozen series. The difference in the frequency of PTC3-positive tumours is more likely to reflect either the technical difficulty of analysing paraffin material or possibly a change in the molecular biology of post-Chernobyl carcinoma with time; the paraffin series were from patients operated in 1990–1994, and the frozen material was from patients operated in 1995 and 1996. A recent study by Smida et al (1999) using frozen samples only, suggests that the ratio of PTC3:PTC1 drops with time post-Chernobyl. However, this report does not provide detailed histological analysis, so we do not know whether these changes correlate with changes in the morphology of tumours studied in the two time periods. Other studies have shown that PTC1 rearrangement is more common in papillary carcinoma from adults with a radiation history (Bounacer et al, 1997), but whether this is related to age of patient at exposure to radiation or the histological subtype of papillary carcinoma warrants further investigation. Only one of our cases showed both PTC1 and -3 rearrangement, contrasting with a recent study using a similar technique (RT-PCR followed by Southern hybridization) which found that multiple rearrangements were not infrequent in papillary carcinomas in adults (Sugg et al, 1998).

TSH receptor pathway mutations are common in hyperfunctioning follicular tumours, but have rarely been found in papillary carcinomas (Challeton et al, 1995; Ohno et al, 1995; Parma et al, 1995; Waldman and Rabes, 1997). p53 mutations have been largely confined to undifferentiated thyroid carcinomas (Ito et al, 1992). There have been two recent reports suggesting that germline polymorphisms of the p53 gene in exon 6 and intron 6 may be found occasionally in the Belarussian population (Hillebrandt et al, 1997; Smida et al, 1997); one study failed to demonstrate oncogenic p53 mutations in post-Chernobyl papillary carcinomas (Suchy et al, 1998), while another found them in only 6% (Nikiforov et al, 1996). Our findings therefore suggest that the pattern of oncogene involvement in the Chernobyl-related cancers correlates with the morphological type, and shows a similar frequency to that found in studies of thyroid cancer generally.

The lack of any *ras* gene mutation needs discussion. Most earlier studies used slot blot and mutation specific oligonucleotide hybridization and found a significant proportion, up to 62%, of papillary carcinomas with a *ras* mutation (Suarez et al, 1990). The difference from our findings could be related to age, environmental or genetic factors, or to the techniques used. One study using Southern blotting and hybridization (Shi et al, 1991) and another using PCR and direct sequencing (Manenti et al, 1994) failed to find any *ras* mutations in papillary carcinomas. Slot blot techniques could be detecting mutations in tumour subpopulations, while PCR and sequencing techniques may fail to detect mutations because of the presence of excess stromal tissue. Our techniques used careful microdissected tumour samples, detected ras mutations in the expected proportion of pancreatic and thyroid follicular carcinomas and reliably detected *ras* mutations if they were present in more than 40% of the tumour cells (data not shown). In agreement with our findings, Suchy et al (1997) found no *ras* mutations in post-Chernobyl childhood thyroid carcinomas. One previous study has suggested that Ki-*ras* mutation in follicular carcinomas may be related to radiation exposure (Wright et al, 1991) and another that it may occasionally occur in radiation-associated papillary carcinomas (Challeton et al, 1995). In a separate study of 26 UK childhood papillary carcinomas, we found no mutations in any of the three ras genes (data not shown). It would therefore appear that ras is not involved in childhood papillary carcinomas whether they are of radiation aetiology or not. We conclude that in follicular carcinogenesis point mutation in a *ras* gene can be an early event, as it has been identified in adenomas as well as carcinomas (Lemoine et al, 1989; Suarez et al, 1990; Wright et al, 1991). In papillary carcinogenesis rearrangement of the *RET* gene is an early event, as immunohistochemistry shows *RET* gene overexpression in a proportion of both small and large papillary carcinomas (Viglietto, 1995), and there is no morphological evidence of any precursor lesion for papillary carcinoma.

While papillary carcinomas are associated with *RET* rearrangements and follicular carcinomas with *ras* mutations, in neither case are these present in all tumours. Other point mutations, including mutations in gsp, and TSHr have been found in follicular tumours (Challeton et al, 1995; Parma et al, 1995; Russo et al, 1995). In papillary tumours rearrangements of the trk oncogene have been found in a small minority of cases (Grieco et al, 1992; Beimfohr et al, 1999), two further rearrangements of the *RET* oncogene have recently been defined in post-Chernobyl thyroid cancer (Fugazzola et al, 1996; Klugbauer et al, 1998), and it is likely that the majority, possibly the great majority of papillary carcinomas are associated with a chromosome rearrangement. Among mutagens radiation is particularly prone to give double-strand DNA breaks leading to deletions and rearrangements (Sankaranarayanan, 1991), lesions that form only a minority of chemical mutagen-induced or naturally occurring mutations. Irradiation of cultured thyroid tumour cells has been shown to be capable of causing *RET* rearrangements (Ito et al, 1993).

We suggest that the restriction of the thyroid tumour increase so far seen to one type of carcinoma is not related to the ability of radiation to cause one specific mutation, but to the general ability of radiation to induce gene rearrangements; those arrangements

 *© 2000 Cancer Research Campaign*

that are oncogenic to the thyroid will lead to clonal expansion. In this case rearrangements that activate the RET or trk tyrosine kinases can stimulate growth and lead to papillary carcinoma, the particular type of thyroid carcinoma found. There is as yet no convincing evidence that radiation is specifically linked to a particular type of *RET* rearrangement, and different selection factors for different *RET* rearrangements could operate at different ages. The relative lack of follicular carcinomas may be linked to the association of the early stages of follicular carcinogenesis with point mutations rather than gene rearrangements, or to a longer latent period, perhaps related to progression through an adenoma.

There is an interesting parallel with the situation in leukaemia, where the main type linked with radiation exposure, chronic myeloid leukaemia, is associated with an abnormal karyotype with a 9:22 translocation in virtually every case, while in chronic lymphatic leukaemia, not known to be related to exposure to radiation the majority of typical cases lack any detectable karyotypic abnormality (Woessner et al, 1996).

Our findings show that there is no doubt that a major increase of childhood thyroid carcinoma has occurred in the area around Chernobyl, and that it is currently restricted to one type of differentiated tumour. Molecular biology investigations correlate with the morphology, and show that papillary carcinoma can be regarded as especially associated with gene rearrangement while follicular carcinoma is associated with point mutation. We suggest that the current restriction of the increase to papillary carcinoma is linked to the particular effectiveness of radiation in causing doublestrand breaks and gene rearrangements.

### ACKNOWLEDGEMENTS

This work was supported by EC contracts numbers COSU CT93 0049, COSU CT CT94 0089 and FI4C CT96 003. We gratefully acknowledge the help of Mrs B Wilson, Mrs S Davies and Mrs H Cook for preparation of the histological material.

### REFERENCES

Baverstock K, Egloff B, Pinchera A, Ruchti C and Williams D (1992) Thyroid cancer after Chernobyl. *Nature* **359**: 21–22

Beimfohr C, Klugbauer S, Demidchik EP, Lengfelder E and Rabes HM (1999) NTRK1 rearrangement in papillary thyroid carcinomas of children after the Chernobyl reactor accident. *Int J Cancer* **80**: 842–847

Bogdanova T, Bragarnik M, Tronko ND, Harach HR, Thomas GA and Williams ED (1996) The pathology of thyroid cancer in Ukraine post-Chernobyl. In: *The Radiological Consequences of the Chernobyl Accident*, Karaoglou A, Desmet G, Kelly GN and Menzel HG (eds), pp. 785–789. European Commission EUR 16544 EN

Bounacer A, Wicker R, Caillou B, Cailleux AF, Sarasin A, Sclumberger M and Suarez HG (1997) High prevalence of activating ret proto-oncogene rearrangements from patients who had received external radiation. *Oncogene* **15**: 1263–1273

Challeton C, Bounacer A, Du Villard JA, Caillou B, De Vathaire F, Monier R, Schlumberger M and Suarez HG (1995) Pattern of ras and gsp oncogene mutations in radiation-associated human thyroid tumors. *Oncogene* **11**: 601–603

Cherstvoy E, Pozcharskaya V, Harach HR, Thomas GA and Williams ED (1996) The pathology of childhood thyroid carcinoma in Belarus. In: *The Radiological Consequences of the Chernobyl Accident*, Karaoglou A, Desmet G, Kelly GN and Menzel HG (eds), pp. 779–784. European Commission EUR 16544 EN

Fuggazzola L, Pilotti S, Pinchera A, Vorontsova TV, Mondellini P, Bongarzone I, Greco A, Astakhova L, Butti MG, Demidchik EP, Pacini F and Pierotti MA (1995) Oncogenic rearrangements of the ret proto-oncogene in papillary thyroid carcinomas from children exposed to the Chernobyl nuclear accident. Cancer Res **55**: 5617–5620

Grieco A, Pierotti MA, Bongarzone I, Pagliardini J, Lanzi C and Della Porta G (1992) Trk-T1 is a novel oncogene formed by the fusion of TPR and Trk genes in human papillary cancer. *Oncogene* **7**: 237–242

Harach HR and Williams ED (1995) Childhood thyroid cancer in England and Wales. *Br J Cancer* **72**: 777–783

Hillebrandt S, Streffer C, Demidchik EP, Biko J and Reiners C (1997) Polymorphisms in the p53 gene in thyroid tumours and blood samples of children from areas in Belarus. *Mutat Res* **381**: 201–207

Ito T, Seyama T, Mizuno T, Tsuyama N, Hayashi T, Hayashi Y, Dohi K, Nakamura N and Akiyama M (1992) Unique association of p53 mutations with undifferentiated but not with differentiated carcinomas of the thyroid gland. *Cancer Res* **52**: 1369–1371

Ito T, Seyama T, Iwamoto KS, Hayashi T, Mizuno T, Tsuyama N, Dohi K, Nakamura N and Akiyama M (1993) In vitro irradiation is able to cause RET oncogene rearrangement. *Cancer Res* **53**: 2940–2943

Ito T, Seyama T, Iwamoto KS, Mizuno T, Tronko ND, Komissarenko IV, Cherstvoy ED, Satow Y, Takiechi N, Dohi K and Akiyama M (1994) Activated ret oncogene in thyroid cancers of children from areas contaminated by Chernobyl accident. *Lancet* **344**: 259

Jacob P, Goulko G, Heidenrich WF, Likhtarev I, Kairo I, Tronko ND, Bogdanora TI, Kenigsberg J, Buglova E, Drozdovitch V, Goloneva A, Demidchik EP, Balanov M, Zvonova I and Beral V (1998) Thyroid risk to children calculated. *Nature* **392**: 31–32

Jhiang SM, Sagartz JE, Tong Q, Parker TJ, Capen CC, Cho JY, Xing S and Ledent C (1996) Targetted expression of the ret/PTC1 oncogene induces papillary thyroid carcinomas. *Endocrinology* **137**: 375–378

Kazakov VS, Demidchik EP and Astakhova LN (1992) Thyroid cancers after Chernobyl. *Nature* **359**: 21

Klugbauer S, Lengfelder E, Demidchik EP and Rabes HM (1995) High prevalence of ret rearrangement in thyroid tumors of children from Belarus after the Chernobyl reactor accident. *Oncogene* **11**: 2459–2467 5620

Klugbauer S, Demidchik EP, Lengfelder E and Rabes HM (1998) Detection of a novel type of ret rearrangement (PTC5) in thyroid carcinomas after Chernobyl and analysis of the involved ret-fused gene RFG5. *Cancer Res* **58**: 198–203

Lemoine NR, Mayall ES, Wyllie FS, Williams ED, Goyns M, Stringer BMJ and Wynford-Thomas D (1989) High frequency of ras oncogene activation in all stages of human thyroid tumorigenesis. *Oncogene* **2**: 159–164

Likhtarev IA, Sobolev BG, Kairo IA Tronko ND, Bogdanova TI, Olenic VA, Epshtein EV and Beral V (1995) Thyroid cancer in Ukraine. *Nature* **375**: 365

Lohmann D, Putz B, Reich U, Bohm J, Prauer H and Hofler H (1993) Mutational spectrum of the p53 gene in human small-cell lung cancer and relationship to clinicopathological data. *Am J Pathol* **142**: 907–915

Manenti G, Pilotti S, Re FC, Della Porta G and Pierotti MA (1994) Selective activation of ras oncogenes in follicular and undifferentiated thyroid carcinomas. *Eur J Cancer* **30A**: 987–993

Nikiforov YE, Nikiforova MN, Gnepp DR and Fagin JA (1996) Prevalence of mutations of ras and p53 in benign and malignant thyroid tumors from children exposed to radiation after the Chernobyl nuclear accident. *Oncogene* **13**: 687–693

Nikiforov YE, Rowland JM, Bove KE, Monforte-Munoz H and Fagin JA (1997) Distinct pattern of ret oncogene rearrangements in morphological variants of radiation-induced and sporadic thyroid papillary carcinomas in children. *Cancer Res* **57**: 1690–1694

Ohno M, Endo T, Ohta K, Gunji K and Onaya T (1995) Point mutations in the thyrotropin receptor in human thyroid tumors. *Thyroid* **5**: 97–100

Parkin DM, Stiller CA, Bieber A, Draper GJ, Terracini B and Young YL (eds) (1988) *International Incidence of Childhood Cancer*. IARC Scientific Publications No 87. International Association for Research an Cancer: Lyon

Parma J, Van Sande J, Swillens S, Tonacchera M, Dumont J and Vassart G (1995) Somatic mutations causing constitutive activity of the thyrotropin receptor are the major cause of hyperfunctioning thyroid adenomas: identification of additional mutations activating both the cyclic adenosine 3′,5′-monophosphate and inositol phosphate-$Ca^{2+}$ cascades. *Mol Endocrinol* **9**: 725–733

Powell DJ, Russell J, Nibu K, Li G, Rhee E, Lioa M, Goldstein M, Keane WM, Santoro M, Fusco A and Rothstein J (1998) The ret/PTC3 oncogene: metastatic solid-type papillary carcinomas in murine thyroids. *Cancer Res* **58**: 5523–5528

Russo D, Arturi F, Schlumberger M, Caillou B, Monier R, Filetti S and Suarez HG (1995) Activating mutations of the TSH receptor in differentiated thyroid carcinomas. *Oncogene* **11**: 1907–1911

Sankaranarayanan K (1991) Ionizing radiation and genetic risks. III. Nature of spontaneous and radiation-induced mutations in mammalian in vitro systems and mechanisms of induction of mutations by radiation. *Mutat Res* **258**: 75–97

© 2000 Cancer Research Campaign 

Santoro M, Carlomango F and Hay ID (1992) Ret oncogene activation in human thyroid neoplasms is restricted to the papillary cancer subtype. *J Clin Invest* **89**: 1517–1522

Santoro M, Chiappetta G, Cerrato A, Salvatore D, Zhang L, Manzo G, Picone A, Portella G, Santelli G, Vecchio G and Fusco A (1996) Development of thyroid papillary carcinomas secondary to tissue-specific expression of the RET/PTC1 oncogene in transgenic mice. *Oncogene* **12**: 1821

Shi YF, Zou MJ, Schmidt H, Juhasz F, Stensky V, Robb D and Farid NR (1991) High rates of ras codon 61 mutation in thyroid tumors in an iodide-deficient area. *Cancer Res* **51**: 2690–2693

Smida J, Zitzelsberger H, Kellerer AM, Lehmann L, Minkus G, Negele T, Spelsberg F, Hieber L, Demidchik EP, Lengfelder E and Bauchinger M (1997) p53 mutations in childhood thyroid tumours from Belarus and in thyroid tumours without radiation history. *Int J Cancer* **73**: 802–807

Smida J, Salassidisk K, Hieber L, Zitelsberger H, Kellere AM, Demidchik EP, Negele T, Spelsberg F, Lengfelder E, Weiner M and Bauchinger M (1999) Distinct frequency of ret rearrangements in papillary thyroid carcinomas of children and adults from Belarus. *Int J Cancer* **80**: 32–38

Suarez HG, du Villard JA, Severino M, Caillou B, Schlumberger M, Tubiana M, Parmentier C and Monier R (1990) Presence of mutations in all three ras genes in human thyroid tumors. *Oncogene* **5**: 565–570

Suchy B, Waldmann V, Klugbauer S and Rabes HM (1998) Absence of RAS and p53 mutations in thyroid carcinomas of children after Chernobyl in contrast to adult thyroid tumours. *Br J Cancer* **77**: 952–955

Sugg SL, Ezzat S, Rosen IB, Freeman JL and Asa SL (1998) Distinct multiple ret/ptc gene rearrangements in multifocal papillary thyroid neoplasia. *J Clin Endocrinol Metab* **83**: 4116–4122

Thomas GA, Bunnell H, Cook HA, Williams ED, Nerovnya A, Cherstvoy ED, Tronko ND, Bogdanova TI, Chiappetta G, Viglietto G, Pentimalli F, Salvatore G, Fusco A, Santoro M and Vecchio G (1999) High prevalence of RET/PTC rearrangements in Ukrainian and Belarussian post-Chernobyl thyroid papillary carcinomas: a strong correlation between RET/PTC3 and the solid/follicular variant. *J Clin Endocrinol Metab* (in press)

Waldmann V and Rabes HM (1997) Absence of Gsα gene mutations in childhood thyroid tumors after Chernobyl in contrast to sporadic adult thyroid neoplasia. *Cancer Res* **57**: 2358–2361

Wright PA, Williams ED, Lemoine NR and Wynford-Thomas D (1991) Radiation-associated and 'spontaneous' human thyroid carcinomas show a different pattern of ras oncogene mutation. *Oncogene* **6**: 471–473

Viglietto G, Chiappetta G, Martinez-Tello FJ, Fukunaga FH, Tallini G, Rigopoulou D, Visconti R, Mastro A, Santoro M and Fusco A (1995) RET/PTC oncogene activation is an early event in thyroid carcinogenesis. *Oncogene* **11**: 1207–1210

Williams ED (1996) Effects on the thyroid in populations exposed to radiation as a result of the Chernobyl accident. In: *One Decade After Chernobyl*. IAEA: 207–230

Williams GH, Rooney S, Thomas GA, Cummins G and Williams ED (1996) RET activation in adult and childhood papillary thyroid carcinoma using a reverse transcriptase-n-polymerase chain reaction approach on archival-nested material. *Br J Cancer* **74**: 585–589

Woessner S, Sole F, Perez-Losada A, Florensa L and Vila RM (1996) Trisomy 12 is a rare cytogenetic finding in typical chronic lymphocytic leukemia. *Leuk Res* **20**: 369–374

 *© 2000 Cancer Research Campaign*
